# The comparison of catastrophic health expenditure and its inequality between urban and rural households in China

**DOI:** 10.1186/s13561-022-00365-z

**Published:** 2022-03-09

**Authors:** Xian-zhi Fu

**Affiliations:** School of Political Science and Public Administration, Wuhan University, Wuhan, 430072 Hubei China

**Keywords:** Catastrophic health expenditure, Inequality, Concentration index, Decomposition

## Abstract

**Background:**

In recent years, the goal of universal coverage of the basic medical insurance schemes has been basically achieved in China, but the heavy economic burden of diseases is still the main cause of poverty in many households. Exploring catastrophic health expenditure (CHE) and its inequality are highly important for forward-looking policymaking. This study aims to compare the incidence, intensity and inequality of CHE between urban and rural households in China.

**Methods:**

This study was based on a national representative household survey—the China Family Panel Studies (CFPS)—that was conducted from 2012 to 2018. Concentration index (CI) was employed to measure the inequality of CHE incidence and overshoot, while the decomposition method of the CI was used to estimate the main influencing factors affecting inequality of CHE incidence.

**Results:**

From 2012 to 2018, the CHE incidence of urban households increased from 11.01 to 11.88%, while the CHE incidence of rural households decreased from 18.42 to 18.31%. During the same period, the CI of CHE incidence for urban households decreased from − 0.1480 to − 0.1693, while that for rural households declined from − 0.1062 to − 0.1501. The major contribution to the pro-poor inequality in CHE incidence was associated with socioeconomic status, lagged CHE, receiving inpatient services, having elderly members, education of household head, and self-assessed health status of household head.

**Conclusions:**

Rural households had higher risk of incurring CHE than urban households. The strong pro-poor inequality for CHE incidence and overshoot could be found in both two groups. The problem of poverty due to illness was more severe among low-income groups in rural areas than in urban areas. The relevant policy interventions should further focus on encouraging the development of supplementary medical insurance and increasing the reimbursement rate for hospitalization expenses in the medical assistance system.

## Background

Universal health coverage (UHC), one of the key targets included in the Sustainable Development Goals (SDG), refers to all people will obtain the essential health services they need without experiencing financial hardship by 2030 [1, 2]. However, a global monitoring report from the WHO shows that in 2017, more than 122 million people globally were classified as “poor” due to health expenditures, and that increasing numbers of individuals were experiencing catastrophic health expenditure (CHE) [3].

One of the fundamental functions of health systems around the world is to improve the ability of households to withstand the financial catastrophe associated with illness [4]. CHE is an indicator reflecting the impact of household health expenditure on household living standards and evaluating the status of financial protection in health system [5, 6]. The occurrence of CHE indicates that health expenditures exceed a certain threshold, and is likely to bring low-income households into poverty, the so-called “poverty caused by illness”.

The Chinese health system has been committed to protecting households from CHE. The Chinese government officially launched the “New Medical Reform” in early 2009, which aims to reduce out-of-pocket (OOP) medical expenditures and achieve universal coverage of essential healthcare by 2020 [7, 8]. Additionally, the establishment of the basic medical insurance system is an effective means of protecting households from CHE. In 1998, the Urban Employee Basic Medical Insurance (UEBMI) was implemented, which provided policy benefits for the employed urban residents to use health services [9]. For all rural residents, an insurance scheme called the New Rural Cooperative Medical Scheme (NRCMS) was piloted from 2003 [10]. In 2010, targeting for all urban residents not covered by the UEBMI, the Urban Residents Basic Medical Insurance (URBMI) was introduced nationwide [11]. In 2013, the coverage rate of basic medical insurance schemes in China exceeded 95%, indicating that the goal of universal coverage of the basic medical insurance schemes was basically achieved [12, 13]. Meanwhile, Chinese government put the development of supplementary medical insurance on the agenda to meet multi-level demand of health care. Specifically, supplementary medical insurance was the supplementary form of basic medical insurance, which included commercial medical insurance, public servant medical subsidy, enterprise supplementary medical subsidy, employee medical subsidy for large medical expenses, and employee mutual medical insurance [14]. In theory, medical insurance could protect households with patients from CHE by reducing OOP medical expenditure. The mutual-aid principle of medical insurance could also alleviate pro-poor inequality in the distribution of CHE by making policy benefits available to more low-income households.

However, evidence indicated that medical expenditure played an important role in the main causes of poverty in Chinese households, especially for rural households [15, 16]. Zhao (2019) observed that the incidence of CHE among rural households in China was as high as 17.70% in 2016 [17]. At the same time, the urbanization rate increased from 51.83% in 2011 to 63.89% in 2020 [18]. The high-speed development of urbanization in China may also lead to the migration of a flood of low-income group, potentially resulting in the occurrence of CHE. Therefore, it is necessary to pay attention to the current situation of CHE of urban and rural households in China. More importantly, the indicators related to the CHE are expressed as proportions, which only reflects the average level of the entire sample. The concentration index (CI), an indicator employed to measure the degree of inequality, could capture the distribution of CHE in different income subgroups. Hence, measuring the CI of CHE is also important for forward-looking policymaking.

Previous studies on CHE around the world have focused on measuring the incidence and inequality of CHE among vulnerable groups, and verifying the impact of relevant policy interventions on CHE. Evidence from Bangladesh, India and South Korean indicated that households with members suffering from chronic diseases were at high risk of incurring CHE [19–21]. Yazdi-Feyzabadi (2019) confirmed that Iran’s Health Transformation Program (HTP) had no considerable success in improving the pro-poor inequality for CHE [6].

In terms of the types of issues explored, the studies that have been conducted on China were also mainly concerned with the two aspects mentioned above. Xu (2015) identified that there was a strong pro-poor inequity of CHE in the rural areas of Shanxi province [22]. Yang (2016) observed that the empty-nest households were at higher risk for CHE than non-empty-nest in Shandong province [23]. Guo (2016) found that NRCMS had a limited role to play in alleviating the inequity for CHE in rural China [24]. Li (2019) verified that critical illness insurance decreased the CHE incidence but increased the intensity of CHE in Jiangsu province [25]. In contrast to other countries, most of the previous studies in China explored the issue of CHE at the provincial level rather than national level. Meanwhile, few of them focused on the disparity of CHE between urban and rural households.

Based on the national representative data in China, this study aimed to measure the CHE incidence, intensity and inequality of urban and rural households from 2012 to 2018, and to analyze the main determinants leading to the unfairness, and to provide policy implications for health system reform in China.

## Methods

### Data source

**Fig. 1:**
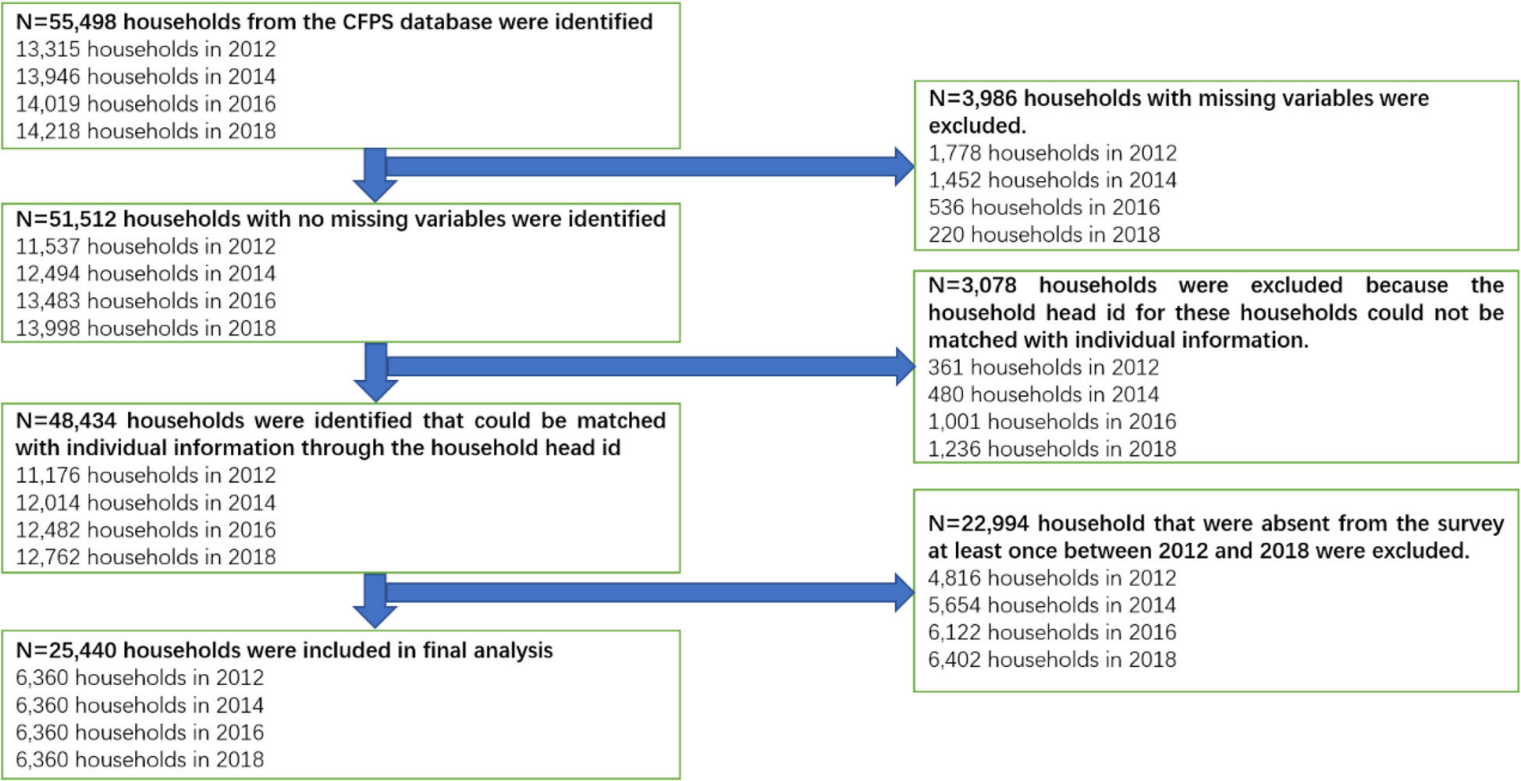
Flow chart of sample selection

This research was based on the raw data from the four waves of China Family Panel Studies (CFPS) conducted between 2012 and 2018. CFPS is a national representative survey directed by the Institute of Social Science Survey (ISSS) of Peking University every 2 years from 2010 to 2018. The survey mainly involved a wide range of information including family socioeconomic status, family relationships, work and income, health status and demography characteristics, etc. A three-stage, stratified, probability-proportional-to-scale (PPS) sampling technique was adopted to select interviewed households from 25 provinces in China. The cases with missing values and logic error on the investigated variables were excluded. Meanwhile, we also excluded the cases that were investigated less than four times from 2012 to 2018. Finally, 6360 households from each round of the survey, including 2761 urban households and 3599 rural households, were included in this study. The detailed sampling process is presented in Fig. 1.

### Measurement of CHE

In this study, CHE was set as the dependent variable. However, there was no consensus on the measure of CHE [26]. To be specific, one strand of literature chose total household expenditure as an indicator of household’s capacity to pay [27, 28], while other studies employed non-food household expenditure instead of total household expenditure [29, 30]. Non-food household expenditure was defined as the total expenditure of a household subtracting the food expenditure of the household. Compared with total household expenditure, non-food household expenditure as the denominator to calculate CHE was more accurate. This was because it partly avoided measurement deviations that were often overlooked in poor households [31, 32]. For the above reasons, and because of the wealth of household data available in the CFPS database, we calculated CHE based on the latter approach. In the previous studies [31], there were many different criteria for defining the threshold of CHE, including: 10, 20, 30, 40%, etc. In order to facilitate comparison with more research results, the threshold for CHE in this study was defined as 40%. Thus, it could be interpreted as households whose OOP medical expenditure that accounted for 40% or more of non-food household expenditure were classified as “households facing CHE”. Since the household questionnaire did not involve information on indirect medical expenditure (e.g., transportation, food, lost productivity due to illness), OOP medical expenditure only included direct medical expenditure in this study. A dummy variable, *E*_*i*_, was defined to determine whether a household experienced CHE, as shown in Eq. (1):

1 $$ {E}_i=\left\{\begin{array}{c}0\  if\ \frac{T_i}{\left({x}_i-{f}_i\right)}< threshold\\ {}1\  if\ \frac{T_i}{\left({x}_i-{f}_i\right)}\ge threshold\end{array}\right. $$

In the Eq. (1), *T*_*i*_ represents the OOP medical expenditure of household *i*, *x*_*i*_ stands for the total expenditure of household *i*, *f*_*i*_ denotes the food expenditure of household *i*, and threshold is equal to 40%. The incidence and intensity of CHE can be calculated by the following Eqs. (2-4):

2 $$ H=\frac{1}{N}\sum \limits_{i=1}^N{E}_i $$

3 $$ O=\frac{1}{N}\sum \limits_{i=1}^N{E}_i\left(\frac{T_i}{\left({x}_i-{f}_i\right)}-z\right) $$

4 $$ MPO=\frac{O}{H} $$

Where N is the total sample size, H stands for the CHE incidence of the households. Intensity of CHE is measured by overshoot and mean positive overshoot (MPO). O represents overshoot, which is defined as the average percentage of OOP medical expenditure that exceeded the given threshold over all households [33]. MPO means the severity of overshoot in the households incurring CHE, and is defined as the average overshoot over all households incurring CHE [29]. The greater value of overshoot and MPO, the heavier economic burden of diseases for the households, and vice versa.

### Definitions of independent variables

Referring to the published literature, a number of variables were incorporated into the random effects panel probit regression model as potential determinants of CHE and its inequality [31, 32, 34, 35]. These independent variables were related to the information of each household surveyed and its household head. Household information included eight variables: lagged CHE, the annual per capita household income, household size, receiving inpatient services, having elderly members, having members with chronic diseases, having members covered by supplementary medical insurance, and geographic location. The information of household head involved nine variables: gender, age, age square, education, marriage, employment status, self-assessed health status, smoking and drinking. Due to the short time series of the database, the lagged value of CHE was set to 2 years. Meanwhile, we used the natural logarithm of the annual per capita household income for regression analysis. All currency-related variables from 2014 to 2018 were deflated to 2012 using the corresponding consumer price index. The details of the independent variables are summarized in Table 1.

**Table 1:** Description of variables

Variables	Description
CHE	The OOP medical expenditure was higher than or equal to 40% of non-food household expenditure; Yes = 1; No = 0
Household expenditure (Yuan)	Total consumption expenditure of a household
OOP medical expenditure (Yuan)	Total out-of-pocket medical expenditure of a household
Food expenditure (Yuan)	Total food consumption expenditure of a household
Lagged CHE	Did the household experience CHE 2 years ago? Yes = 1; No^a^ = 0
Income (Yuan)	The annual per capita household income
Lnincome	The natural logarithm of annual per capita household income
Household size	The number of household members
Inpatient	At least one household member received inpatient services in last year; Yes = 1; No^a^ = 0
Elderly members	At least one household member over 60 years old; Yes = 1; No^a^ = 0
Chronic diseases	At least one household member with chronic diseases; Yes = 1; No^a^ = 0
Supplementary medical insurance	At least one household member covered by supplementary medical insurance; Yes = 1; No^a^ = 0
Geographic location	East^a^ = 1, Central = 2, West = 3
Gender of household head	Female^a^ = 0; male = 1
Age	The age of household head
Education of household head	Illiterate^a^ = 1; Primary school = 2; Middle school = 3; High school and above = 4
Marriage of household head	Married = 1, Unmarried^a^ = 0
Employment status of household head	Employed = 1, Unemployed^a^ = 0
Self-assessed health status of household head	Healthy = 1; Unhealthy^a^ = 0
Smoking	Has the household head smoked in the past month? Yes = 1; No^a^ = 0
Drinking	Has the household head drunk more than three times a week in the past month? Yes = 1; No^a^ = 0

Note: ^a^ Reference group; CHE = Catastrophic health expenditure; OOP = Out-of-pocket medical expenditure

### Methodology

The concentration index (CI), developed by Wagstaff and Van Doorslaer, was the indicator most commonly used to measure the inequity of CHE [36, 37]. The CI can reflect the situation of all sample households from socioeconomic dimension, and is sensitive to the changes in the distribution of the households across socioeconomic groups [38, 39]. The concentration curve depicts the cumulative percentage of the households, ranked by household income from the poorest to the richest (x-axis), against the cumulative percentage of CHE (y-axis), while the CI is defined as twice the area enclosed by the concentration curve and absolute fairness line [36, 37, 40]. The value of the CI ranges from − 1 to + 1, and the smaller the absolute value of the CI is, the more fair is [38]. When the CI is equal to zero, the distribution of CHE is absolutely fair [29, 41]. A positive CI represents that the distribution of CHE is more conducive to the richer households, and vice versa [42].

The calculation of the CI is shown in Eq. (5):

5 $$ \mathrm{CI}=\frac{2}{\upmu}{\operatorname{cov}}_w\left({\mathrm{y}}_i,{\mathrm{r}}_i\right) $$

where y_*i*_ denotes the relevant indicators for CHE, r_*i*_ is the fractional rank of the households in terms of income distribution and μ represents the mean of CHE.

Given the differences in opportunity costs, we modify the CHE incidence and overshoot by giving greater weights to poorer households [28]. The specific calculations are based on the following two equations:

6 $$ {H}^W=H\left(1-{CI}_H\right) $$

7 $$ {O}^W=O\left(1-{CI}_O\right) $$

Where *CI*_*H*_ is the CI of CHE incidence, and *CI*_*O*_ denotes the CI for the overshoot.

As proposed by Wagstaff et al., the decomposition of CI was employed in this study to analyze the contribution of relevant independent variables to the inequality of CHE incidence [36]. The probit regression model was established to decompose the CI in this study. As the probit model is a non-linear model, the linear approximation to the non-linear model is calculated by estimating the marginal effect evaluated at the covariate means [36]. The specific regression model can be expressed as:

8 $$ {\mathrm{y}}_i=\updelta +{\sum}_k{\upgamma}_{\mathrm{k}}{\mathrm{z}}_{ki}+{\upvarepsilon}_i $$

In the Eq. (8), y_*i*_ is whether the household has incurred CHE, z_*k*_ stands for the independent variable, and δ, γ_k_ and ε denote the constant term, marginal effect and disturbance term, respectively.

The method of decomposition of CI can be specified as:

9 $$ \mathrm{CI}={\sum}_{\mathrm{k}}\frac{\upgamma_{\mathrm{k}}{\overline{\mathrm{z}}}_{\mathrm{k}}}{\upmu}{\mathrm{C}}_{\mathrm{k}}+\frac{GC_{\varepsilon }}{\upmu} $$

Where ${\overline{\mathrm{z}}}_{\mathrm{k}}$ denotes the mean of each independent variable, C_k_ represents the CI of each independent variable, $\left({\upgamma}_k{\overline{\mathrm{z}}}_{\mathrm{k}}/\upmu \right)$ is the elasticity of CI, and (*GC*_*ε*_/μ) stands for the error term [33].

All analyses were performed in STATA software version 15.1.

## Results

### Descriptive statistics

Summary statistics regarding each variable of the urban and rural households is reported in Table 2. The average annual household consumption in urban areas rose from 45,150 YUAN in 2012 to 63,918 YUAN in 2018, while that in rural areas increased from 30,879 YUAN in 2012 to 37,587 YUAN in 2018. The household size in urban areas decreased from 3.60 in 2012 to 3.51 in 2018, while the household size in rural areas also showed a declining trend over the same period.

**Table 2:** Summary statistics of variables in urban and rural households

Variables	2012				2014			
	Urban households		Rural households		Urban households		Rural households	
	Mean(N)	S.D. (%)	Mean(N)	S.D. (%)	Mean(N)	S.D. (%)	Mean(N)	S.D. (%)
Sample size	2761	100%	3599	100%	2761	100%	3599	100%
Household expenditure (Yuan)	45,150	54,696	30,879	32,972	53,849	53,201	33,829	41,199
OOP medical expenditure (Yuan)	3488	10,495	3581	11,904	4670	15,460	4273	12,341
Food household expenditure (Yuan)	16,911	14,396	13,060	12,070	20,034	14,869	10,084	9770
Income (Yuan)	16,721	22,577	9412	13,186	19,515	32,466	10,190	18,559
Lnincome	9.23	1.14	8.64	1.16	9.39	1.09	8.68	1.21
Household size	3.60	1.54	4.21	1.82	3.59	1.60	4.18	1.83
Inpatient								
Yes	649	23.51	883	24.53	786	28.47	1000	27.79
No^a^	2112	76.49	2716	75.47	1975	71.53	2599	72.21
Elderly members								
Yes	998	36.15	1360	37.79	1225	44.37	1703	47.32
No^a^	1763	63.85	2239	62.21	1536	55.63	1896	52.68
Chronic diseases								
Yes	793	28.72	977	27.15	1053	38.14	1284	35.68
No^a^	1968	71.28	2622	72.85	1708	61.86	2315	64.32
Supplementary medical insurance								
Yes	71	2.57	47	1.31	125	4.53	50	1.39
No^a^	2690	97.43	3552	98.69	2636	95.47	3549	98.61
Geographic location								
East^a^	1370	49.62	1260	35.01	1370	49.62	1260	35.01
Central	902	32.67	1058	29.40	902	32.67	1058	29.40
West	489	17.71	1281	35.59	489	17.71	1281	35.59
Gender of household head								
Female^a^	1450	52.52	1442	40.07	1482	53.68	1493	41.48
Male	1311	47.48	2157	59.93	1279	46.32	2106	58.52
Age	49.78	12.99	49.60	12.18	51.33	12.75	51.37	12.06
Age square	2647.14	1334.76	2608.19	1224.73	2797.86	1349.55	2783.98	1254.45
Education of household head								
Illiterate^a^	468	16.95	1227	34.09	467	16.91	1220	33.90
Primary school	523	18.94	1007	27.98	515	18.65	1030	28.62
Middle school	883	31.98	981	27.26	880	31.87	977	27.15
High school and above	887	32.13	384	10.67	899	32.56	372	10.34
Marriage of household head								
Married	2450	88.74	3281	91.16	2420	87.65	3262	90.64
Unmarried^a^	311	11.26	318	8.84	341	12.35	337	9.36
Employment status of household head								
Employed	1773	64.22	3115	86.55	1801	65.23	3136	87.14
Unemployed^a^	988	35.78	484	13.45	960	34.77	463	12.86
Self-assessed health status of household head								
Healthy	1663	60.23	2085	57.93	1865	67.55	2280	63.35
Unhealthy^a^	1098	39.77	1514	42.07	896	32.45	1319	36.65
Smoking								
Yes	776	28.11	1472	40.90	738	26.73	1372	38.12
No^a^	1985	71.89	2127	59.10	2023	73.27	2227	61.88
Drinking								
Yes	474	17.17	672	18.67	438	15.86	677	18.81
No^a^	2287	82.83	2927	81.33	2323	84.14	2922	81.19
Variables	2016				2018			
	Urban households		Rural households		Urban households		Rural households	
	Mean(N)	S.D. (%)	Mean(N)	S.D. (%)	Mean(N)	S.D. (%)	Mean(N)	S.D. (%)
Sample size	2761	100%	3599	100%	2761	100%	3599	100%
Household expenditure (Yuan)	65,048	106,127	36,853	44,010	63,918	63,518	37,587	40,074
OOP medical expenditure (Yuan)	5517	17,708	4912	17,525	5234	12,473	4901	11,932
Food household expenditure (Yuan)	21,873	19,667	10,674	11,175	23,003	17,268	11,166	12,336
Income (Yuan)	27,401	60,884	12,937	66,828	32,762	62,048	13,758	17,529
Lnincome	9.72	0.95	8.97	0.93	9.96	0.93	9.12	0.94
Household size	3.59	1.65	4.16	1.93	3.51	1.72	3.97	1.92
Inpatient								
Yes	844	30.57	1107	30.76	878	31.80	1214	33.73
No^a^	1917	69.43	2492	69.24	1883	68.20	2385	66.27
Elderly members								
Yes	1330	48.17	1835	50.99	1451	52.55	2023	56.21
No^a^	1431	51.83	1764	49.01	1310	47.45	1576	43.79
Chronic diseases								
Yes	1026	37.16	1315	36.54	993	35.97	1369	38.04
No^a^	1735	62.84	2284	63.46	1768	64.03	2230	61.96
Supplementary medical insurance								
Yes	157	5.69	105	2.92	137	4.96	84	2.33
No^a^	2604	94.31	3494	97.08	2624	95.04	3515	97.67
Geographic location								
East^a^	1369	49.58	1260	35.01	1372	49.69	1259	34.98
Central	904	32.74	1058	29.40	902	32.67	1057	29.37
West	488	17.67	1281	35.59	487	17.64	1283	35.65
Gender of household head								
Female^a^	1499	54.29	1550	43.07	1456	52.73	1564	43.46
Male	1262	45.71	2049	56.93	1305	47.27	2035	56.54
Age	53.08	12.97	52.78	12.42	54.65	12.96	54.52	12.41
Age square	2986.08	1410.88	2940.31	1318.79	3154.92	1452.19	3126.48	1356.12
Education of household head								
Illiterate^a^	451	16.33	1184	32.90	401	14.52	1132	31.45
Primary school	541	19.59	1041	28.92	508	18.40	1006	27.95
Middle school	854	30.93	971	26.98	881	31.91	1056	29.34
High school and above	915	33.14	403	11.20	971	35.17	405	11.25
Marriage of household head								
Married	2389	86.53	3206	89.08	2362	85.55	3136	87.14
Unmarried^a^	372	13.47	393	10.92	399	14.45	463	12.86
Employment status of household head								
Employed	1747	63.27	3085	85.72	1710	61.93	3017	83.83
Unemployed^a^	1014	36.73	514	14.28	1051	38.07	582	16.17
Self-assessed health status of household head								
Healthy	1694	61.35	2148	59.68	1811	65.59	2225	61.82
Unhealthy^a^	1067	38.65	1451	40.32	950	34.41	1374	38.18
Smoking								
Yes	716	25.93	1280	35.57	721	26.11	1324	36.79
No^a^	2045	74.07	2319	64.43	2040	73.89	2275	63.21
Drinking								
Yes	445	16.12	645	17.92	456	16.52	610	16.95
No^a^	2316	83.88	2954	82.08	2305	83.48	2989	83.05

Note: ^a^ Reference group; OOP medical expenditure = out-of-pocket medical expenditure

Compared with urban households, the rural households had higher probability in receiving inpatient services in the last 12 months, having elderly members and having married household head. The coverage rate of supplementary medical insurance for urban households was higher than that for rural households. In urban areas, more than half of household heads were female, while in rural areas the opposite was true. The education level of household heads in urban areas was mainly concentrated in high school and above, while the highest proportion of household heads in rural areas had almost no education. In addition, rural household heads had a higher percentage of smoking and drinking than urban household heads.

### CHE and the inequality for CHE

Table 3 illustrates CHE incidence, intensity and inequality of urban and rural households. The concentration curves of CHE incidence and overshoot are shown in Figs. 2, 3, 4, 5, 6, 7, 8 and 9.

**Table 3:** CHE incidence, intensity and inequality for urban and rural households

		CHE incidence and intensity					Inequality in CHE	
		Incidence	H^W^	Overshoot	O^W^	MPO	CI_H_	CI_O_
2012	Urban households	11.01%	12.64%	2.15%	2.51%	19.53%	−0.1480	−0.1694
	Rural households	18.42%	20.38%	4.02%	4.57%	21.82%	−0.1062	− 0.1373
2014	Urban households	11.99%	13.68%	2.51%	2.99%	20.93%	−0.1409	− 0.1903
	Rural households	16.64%	18.74%	3.55%	4.05%	21.33%	−0.1261	− 0.1399
2016	Urban households	12.42%	13.85%	2.53%	2.89%	20.37%	−0.1148	− 0.1431
	Rural households	18.45%	20.88%	4.07%	4.75%	22.06%	−0.1316	− 0.1659
2018	Urban households	11.88%	13.89%	2.34%	2.74%	19.70%	− 0.1693	−0.1707
	Rural households	18.31%	21.06%	3.85%	4.54%	21.03%	−0.1501	− 0.1782

Note: *CHE* Catastrophic health expenditure, *H*^*W*^ rank-weighted catastrophic health expenditure incidence, *O*^*W*^ rank-weighted overshoot, *MPO* Mean positive overshoot; *CI*_*H*_ the concentration index of catastrophic health expenditure incidence; *CI*_*O*_ the concentration index of overshoot

**Fig. 2:**
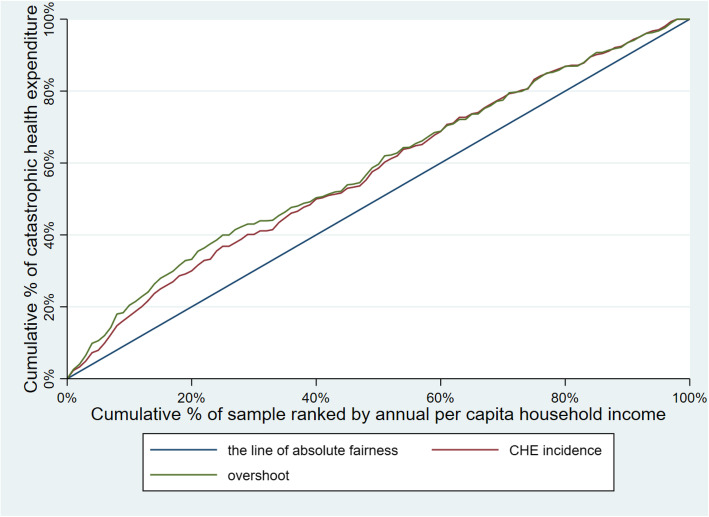
Concentration curves of CHE incidence and overshoot in urban households, China, 2012

**Fig. 3:**
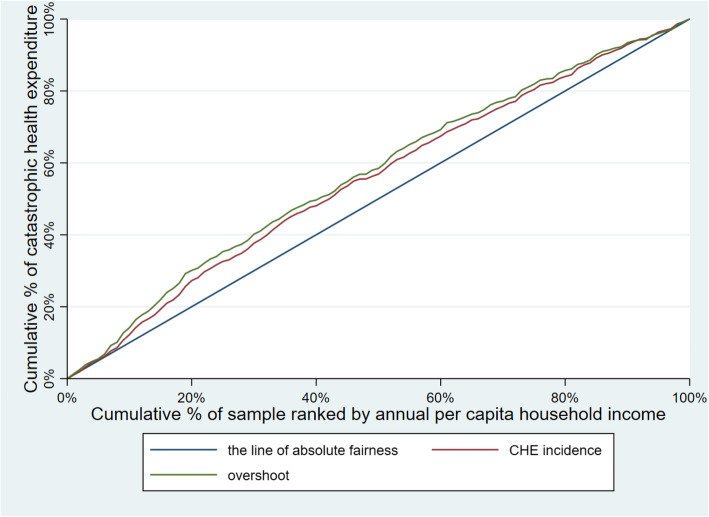
Concentration curves of CHE incidence and overshoot in rural households, China, 2012

**Fig. 4:**
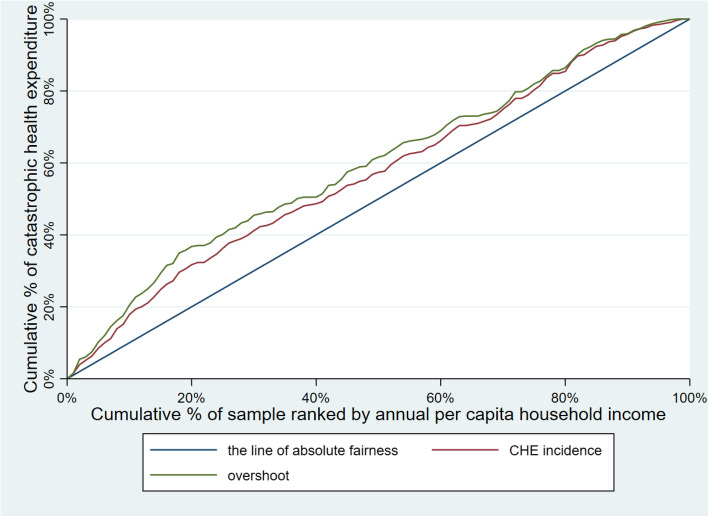
Concentration curves of CHE incidence and overshoot in urban households, China, 2014

**Fig. 5:**
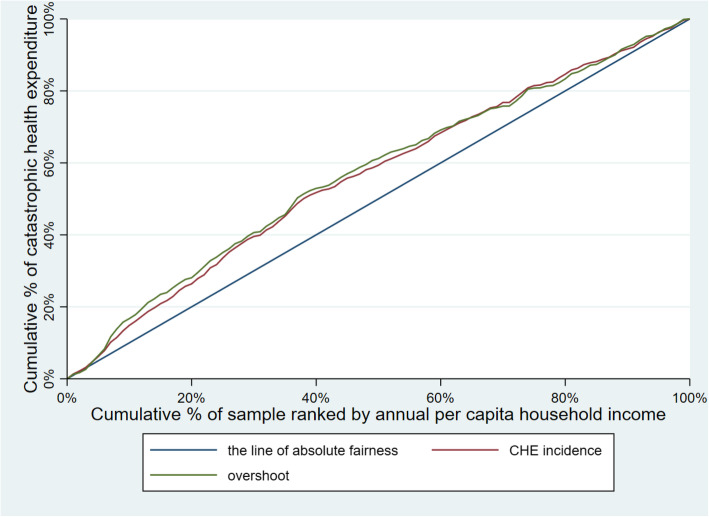
Concentration curves of CHE incidence and overshoot in rural households, China, 2014

**Fig. 6:**
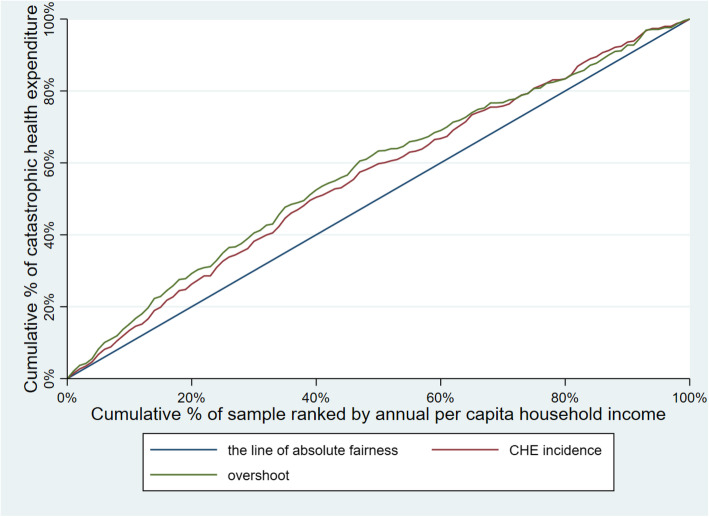
Concentration curves of CHE incidence and overshoot in urban households, China, 2016

**Fig. 7:**
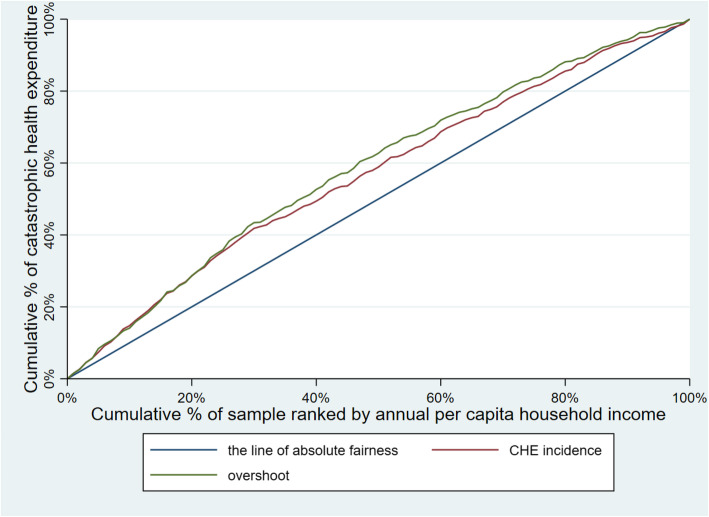
Concentration curves of CHE incidence and overshoot in rural households, China, 2016

**Fig. 8:**
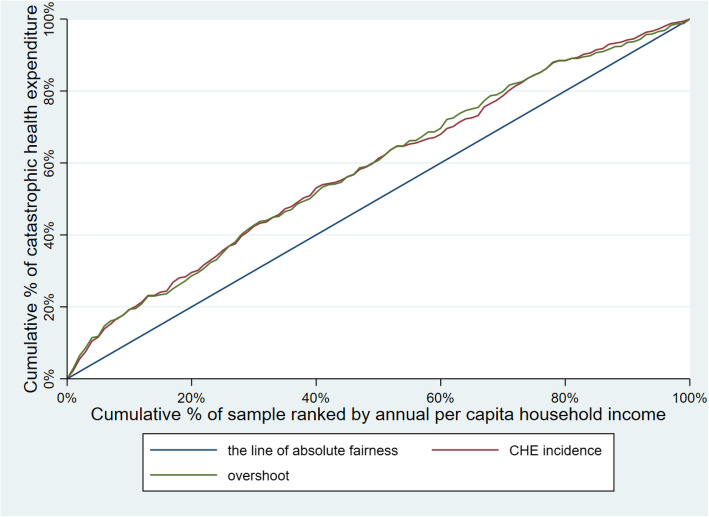
Concentration curves of CHE incidence and overshoot in urban households, China, 2018

**Fig. 9:**
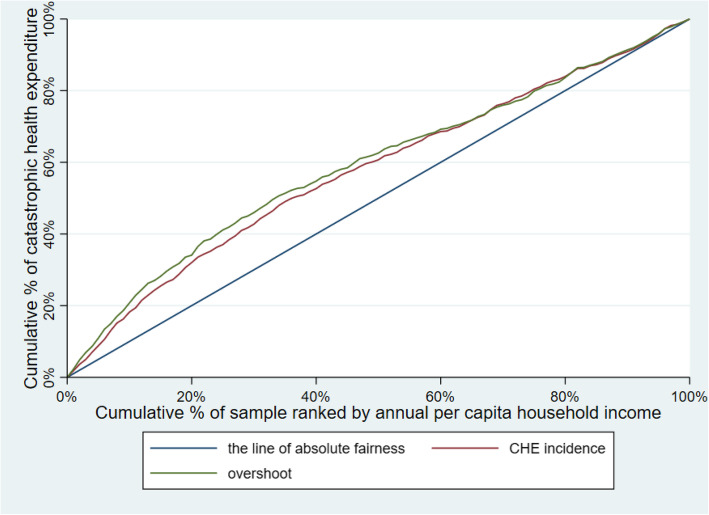
Concentration curves of CHE incidence and overshoot in rural households, China, 2018

From 2012 to 2018, the CHE incidence of urban households increased from 11.01 to 11.88%, while the CHE incidence of rural households decreased from 18.42 to 18.31%. Similar trends were found for both overshoot and MPO. The CHE incidence and overshoot increased slightly for both urban and rural households, by giving greater weights to the poorer households. However, none of the above indicators showed a steady upward or downward trend.

The CI for CHE incidence and overshoot were both negative. These results showed an obvious pro-poor inequality in the distribution of CHE incidence and overshoot across socioeconomic groups. Similarly, the concentration curves of CHE incidence and overshoot were both above the line of absolute fairness, which also highlighted the pro-poor inequality of CHE incidence and overshoot (Figs. 2, 3, 4, 5, 6, 7, 8 and 9). From 2012 to 2018, urban household showed an unstable increase in the absolute values of the CI for both the CHE incidence and overshoot. In contrast, the increase of rural households on the corresponding indicators was relatively stable over the same period. In addition, the CHE incidence and overshoot were greater in absolute terms for urban households than for rural households between 2012 and 2014, until the situation reversed in 2016.

### Associated factors of CHE incidence

Table 4 presents the random effects panel probit regression results for CHE incidence.

**Table 4:** Marginal effect of each variable associated with CHE incidence

Variables	Urban households		Rural households	
	dy/dx^b^	Std. Err.	dy/dx^b^	Std. Err.
Lagged CHE	0.0567**	0.0119	0.1144*	0.0567
Lnincome	− 0.0270**	0.0033	− 0.0179*	0.0079
Household size	−0.0142**	0.0022	−0.0221*	0.0090
Inpatient, yes	0.1153**	0.0068	0.1458*	0.0607
Elderly members, yes	0.0117	0.0087	0.0276*	0.0135
Chronic diseases, yes	0.0241**	0.0070	0.0306*	0.0143
Supplementary medical insurance, yes	−0.0547**	0.0187	−0.0441	0.0315
Geographic location				
East^a^				
Central	0.0013	0.0078	−0.0132	0.0099
West	−0.0188	0.0097	−0.0208	0.0120
Gender of household head, male	0.0128	0.0084	0.0117	0.0098
Age	0.0001	0.0018	−0.0023	0.0021
Age square	0.0000	0.0000	0.0001*	0.0001
Education of household head				
Illiterate^a^				
Primary school	−0.0083	0.0103	−0.0198	0.0116
Middle school	−0.0290**	0.0100	−0.0298*	0.0151
High school and above	−0.0343**	0.0109	−0.0270	0.0163
Marriage of household head, married	−0.0033	0.0097	0.0183	0.0130
Employment status of household head, employed	−0.0167*	0.0078	−0.0344*	0.0166
Self-assessed health status of household head, healthy	−0.0447**	0.0069	−0.0552*	0.0238
Smoking, yes	−0.0082	0.0091	0.0004	0.0085
Drinking, yes	−0.0209*	0.0102	−0.0287	0.0150

Note: ^a^ Reference group; ^b^ Marginal effect; * *p* < 0.05; ** *p* < 0.01

As shown in Table 4, economic status and household size were negatively associated with the occurrence of exposure to CHE for both urban and rural households. Education at the middle school and above reduced CHE occurrence. The risk of CHE was also decreased among households with a healthy or employed head. Meanwhile, experiencing CHE 2 years ago, receiving inpatient services and having household members suffering from chronic diseases significantly increased the CHE incidence of urban and rural households. In addition, the likelihood of urban households experiencing CHE was significantly lower when household members were covered by supplementary medical insurance or when the household head had a drinking habit. CHE incidence significantly increased when there were elderly members in rural households.

### Decomposition of inequality in CHE incidence

Table 5 displays the results of estimated absolute and relative contribution for each variable to the inequality of CHE incidence.

**Table 5:** The contribution of each independent variable to the inequality in CHE incidence

Variables	2012				2014			
	Urban households		Rural households		Urban households		Rural households	
	Cont^b^	Per^c^	Cont^b^	Per^c^	Cont^b^	Per^c^	Cont^b^	Per^c^
Lagged CHE	–	–	–	–	−0.0115	8.19%	− 0.0157	12.48%
Lnincome	−0.0974	65.79%	−0.0168	15.82%	−0.1124	79.79%	−0.0551	43.72%
Household size	0.0315	−21.28%	−0.0037	3.45%	0.0181	−12.88%	0.0012	−0.98%
Inpatient, yes	−0.0022	1.47%	−0.0095	8.91%	− 0.0055	3.93%	−0.0072	5.71%
Elderly members, yes	−0.0001	0.08%	−0.0033	3.12%	0.0001	−0.04%	−0.0079	6.26%
Chronic diseases, yes	0.0057	−3.86%	0.0016	−1.54%	0.0019	− 1.38%	− 0.0001	0.09%
Supplementary medical insurance, yes	−0.0014	0.98%	−0.0001	0.06%	−0.0021	1.51%	−0.0010	0.78%
Geographic location								
East^a^								
Central	−0.0059	3.99%	0.0005	−0.45%	0.0001	−0.04%	−0.0013	0.99%
West	−0.0012	0.79%	−0.0003	0.29%	0.0074	−5.22%	0.0060	−4.73%
Gender of household head, male	0.0026	−1.75%	−0.0004	0.36%	0.0001	−0.10%	−0.0008	0.65%
Age	−0.0124	8.38%	0.0157	−14.82%	−0.0001	0.04%	0.0083	−6.58%
Age square	0.0148	−9.98%	−0.0302	28.48%	0.0124	−8.78%	−0.0184	14.55%
Education of household head								
Illiterate^a^								
Primary school	0.0016	−1.06%	0.0017	−1.61%	0.0038	−2.66%	−0.0004	0.34%
Middle school	0.0015	−1.04%	−0.0079	7.43%	0.0014	−1.01%	−0.0045	3.53%
High school and above	−0.0315	21.30%	− 0.0135	12.70%	−0.0146	10.34%	−0.0045	3.59%
Marriage of household head, married	0.0000	0.00%	−0.0001	0.04%	−0.0011	0.75%	−0.0003	0.27%
Employment status of household head, employed	0.0010	−0.68%	− 0.0010	0.96%	0.0044	−3.10%	0.0001	−0.10%
Self-assessed health status of household head, healthy	−0.0093	6.25%	−0.0065	6.12%	−0.0079	5.64%	−0.0080	6.38%
Smoking, yes	0.0009	−0.61%	− 0.0002	0.16%	0.0009	−0.61%	−0.0001	0.03%
Drinking, yes	−0.0004	0.29%	−0.0024	2.23%	−0.0007	0.52%	−0.0035	2.81%
Residual variables	−0.0458	30.94%	− 0.0298	28.29%	−0.0356	25.11%	−0.0129	10.21%
Variables	2016				2018			
	Urban households		Rural households		Urban households		Rural households	
	Cont^b^	Per^c^	Cont^b^	Per^c^	Cont^b^	Per^c^	Cont^b^	Per^c^
Lagged CHE	− 0.0088	7.65%	− 0.0225	17.06%	− 0.0129	7.62%	−0.0225	15.01%
Lnincome	−0.0733	63.88%	−0.0459	34.88%	−0.1321	78.02%	−0.0758	50.49%
Household size	0.0259	−22.58%	0.0106	−8.03%	0.0320	−18.91%	0.0058	−3.90%
Inpatient, yes	−0.0174	15.17%	−0.0154	11.71%	−0.0020	1.19%	−0.0078	5.19%
Elderly members, yes	−0.0001	0.09%	−0.0137	10.38%	−0.0025	1.46%	−0.0048	3.21%
Chronic diseases, yes	0.0009	− 0.80%	−0.0015	1.15%	0.0027	−1.60%	− 0.0025	1.66%
Supplementary medical insurance, yes	−0.0032	2.77%	−0.0011	0.83%	−0.0029	1.72%	−0.0025	1.67%
Geographic location								
Eastt^a^								
Central	0.0024	−2.06%	−0.0005	0.42%	−0.0022	1.30%	0.0001	-0.06%
West	−0.0013	1.09%	0.0010	−0.73%	0.0049	−2.89%	0.0057	−3.81%
Gender of household head, male	0.0011	−0.96%	−0.0011	0.86%	0.0004	−0.24%	0.0000	0.01%
Age	0.0006	−0.52%	0.0149	−11.36%	0.0024	−1.40%	0.0155	−10.32%
Age square	0.0039	−3.36%	−0.0298	22.63%	0.0019	−1.13%	−0.0295	19.63%
Education of household head								
Illiterate^a^								
Primary school	0.0076	−6.63%	−0.0001	0.01%	−0.0058	3.42%	−0.0003	0.17%
Middle school	0.0010	−0.86%	−0.0020	1.57%	0.0026	−1.56%	−0.0055	3.68%
High school and above	−0.0290	25.28%	−0.0033	2.52%	−0.0200	11.80%	−0.0037	2.46%
Marriage of household head, married	0.0002	−0.15%	0.0019	−1.43%	0.0006	−0.35%	0.0030	−1.99%
Employment status of household head, employed	0.0015	−1.31%	−0.0023	1.72%	0.0049	−2.88%	−0.0026	1.71%
Self-assessed health status of household head, healthy	−0.0075	6.51%	−0.0077	5.85%	−0.0085	5.02%	−0.0093	6.20%
Smoking, yes	0.0010	−0.84%	−0.0003	0.24%	−0.0005	0.28%	0.0001	−0.04%
Drinking, yes	−0.0005	0.44%	−0.0008	0.63%	−0.0014	0.80%	−0.0006	0.39%
Residual variables	−0.0198	17.19%	−0.0120	9.09%	−0.0309	18.33%	−0.0129	8.64%

Note: ^a^ Reference group; ^b^ Contribution to concentration index; ^c^ percentage of contribution to concentration index

With regard to the inequality in CHE incidence among urban and rural households, the main contributing factors remained largely unchanged between 2012 to 2018. Specifically, the main contribution to the inequality in CHE incidence among urban households in 2018 was associated with lagged CHE (7.62%), economic status (78.02%), household size (− 18.91%), receiving inpatient services (1.19%), education of household head (high school and above, 11.80%), and self-assessed health status of household head (5.02%). In rural households, the majority of the inequality of incurring CHE was associated with lagged CHE (15.01%), economic status (50.49%), household size (− 3.90%), receiving inpatient services (5.19%), having elderly members (3.21%), education of household head (middle school, 3.68%; high school and above, 2.46%), and self-assessed health status of household head (6.20%). Furthermore, residual variables contributed extensively to the increase in pro-poor inequality of incurring CHE (urban households: 18.33%; rural households: 8.64%). The same logic can be applied to the urban and rural households in 2012/2014/2016.

## Discussion

By deeply analyzing the national representative data, the present study estimates the incidence, intensity and inequality of CHE for urban and rural households in China. Here, we identified that both urban and rural households suffered CHE, with varying incidence and intensity, and that situation did not improve dramatically from 2012 to 2018. We also examined that the rural households had higher probability and intensity of incurring CHE than those of the urban households, which implied that rural households had higher risk of incurring CHE and heavier economic burden of diseases. Xu reported that the CHE incidence of rural areas of Shaanxi Province in 2013 was 15.83% [22]. As Si showed, the occurrence of exposure to CHE for urban households with hypertension in 2013 was 21.50% [43]. The differences between this study and previous researches in CHE incidence could be attributed to the diversities of research samples and areas.

In addition, we found a significant increase of the CHE incidence and overshoot by giving greater weights to the poorer households. It meant that the CHE incidence and overshoot would increase if the opportunity cost difference was considered from the perspective of social welfare in both two groups. In other words, the issue of CHE was worse than it appeared simply by observing the proportion of the households exceeding the threshold (40%), since it ignored the fact that the poorer households were more likely to exceed the threshold [28].

Furthermore, this article identified several key determinants of CHE incidence and most of them were similar with prior studies [14, 43, 44]. As we expected, higher annual per capita household income and better self-rated health status of the household head were both significantly associated with lower CHE incidence. The greater household size was more likely to avoid CHE. The risk of CHE was decreased among urban and rural households if the household head had a high level of education, or if the household head was employed. Conversely, receiving inpatient services and having members suffering chronic diseases may increase the risk of incurring CHE. Moreover, these effects were more prominent in rural households rather than in urban households, which meant that the related policy interventions should give priority to the health needs of vulnerable households, especially in rural areas. The study also found that urban and rural households experiencing CHE 2 years ago were significantly more likely to be impoverished again due to illness. The comparative analysis of the marginal effects also showed that the problem of poverty due to illness was more persistent in rural households than in urban ones.

Supplementary medical insurance significantly reduced the CHE incidence among urban households, but did not significantly affect the CHE incidence among rural households. One of the potential reasons for this phenomenon is that rural households have lower coverage rate of supplementary medical insurance than urban households. The coverage rate of supplementary medical insurance for urban households increased from 2.57% in 2012 to 4.96% in 2018, while that for rural households rose from 1.31% in 2012 to 2.33% in 2018. It implied that the Chinese government should encourage the development of supplementary medical insurance, especially in rural areas, which is conducive to the formation of a multi-dimensional medical insurance system to alleviate the financial burden of rural patients.

There were strong pro-poor inequalities in CHE incidence and overshoot among urban and rural households, and the inequitable situation worsened from 2012 to 2018. This finding was concordant with the result of Sun et al.’ study [45]. The comparative analysis also revealed that rural households showed a greater and more stable increase in the absolute value of CI regarding the CHE incidence and overshoot from 2012 to 2018 compared to urban households. These results indicated that the problem of poverty due to illness was more severe for rural low-income groups than for urban low-income groups.

By decomposing the CI of CHE incidence, this article explored the contribution of each determinant to the inequality of CHE incidence among urban and rural households in China. Economic status made the greatest contribution to the pro-poor inequality of CHE incidence in both two groups, which indicated that the economic status was still the prime factor causing poor households to suffer CHE [22, 32, 43, 46]. The second largest contribution to the pro-poor inequality of CHE incidence, stemming from lagged CHE, reinforced the idea that poor households were more vulnerable to succussive CHE. Meanwhile, the contribution of economic status and lagged CHE to the pro-poor inequality in CHE incidence among urban and rural households increased substantially between 2012 and 2018, indicating a further deterioration of the situation. In view of this problem, the most important goal of policy interventions is to alleviate the gap between the rich and the poor, such as implementing effective measures that improve the economic performance for low-income households. Unlike previous studies [31, 32], our study found that household size made the largest pro-rich contribution to the inequality for CHE incidence in both two groups, which demonstrated that household size reduced the risk of incurring CHE in poor households. This could be explained by the fact that the low-income households in China were associated with greater household size [24, 47], which was beneficial to alleviate the risk of incurring CHE. From 2012 to 2018, the pro-rich contribution of household size to the inequality in CHE incidence did not fluctuate significantly, indicating that the financial protection effect of household size on poor households was relatively stable. The level of education of household head increased the risk of incurring CHE among poor households, especially in urban areas. It further demonstrated that human capital played an important role in household economic protection and the necessity of generally improving citizens’ education level through policy interventions [43].

The contribution of receiving inpatient services to the inequality of experiencing CHE was in a pro-poor direction, increasing the probability of incurring CHE in poor households. It can be attributed to the purchasing power of inpatient services was weaker in poor households than in affluent ones. From 2012 to 2018, there was a small reduction in the pro-poor contribution of receiving inpatient services to the inequality in CHE incidence among urban and rural households, indicating that the current problem has lessened but it is still unsolved. The other factors such as having elderly members, and self-assessed health status of household head, were also the major reasons for the pro-poor inequality of CHE incidence, especially in rural households. In order to solve the above problems, the medical security department should appropriately increase the reimbursement rate of the medical assistance system according to the medical needs of low-income groups, especially for hospitalization expenses.

Additionally, the presence of chronic diseases made a minor pro-poor contribution to the inequality of CHE incidence in both two groups. Given the current significant impact of chronic diseases on the economic burden of diseases, this result was lower than our expectations. Based on Eq. (9), the contribution of each variable is equal to the product of the elasticity of the corresponding variable and the CI of the corresponding variable. Therefore, the minor contribution of chronic diseases can be explained by a small elasticity of chronic diseases in both urban and rural households.

There are several limitations in this study. Firstly, the negative contributions of residual variables indicate that the omission of related variables (e.g., outpatient services utilization, distance to the nearest health facilities, the levels of medical facilities) leads to some unexplained contribution of pro-poor inequality owing to the data availability. Secondly, the present research uses a conservative method to estimate the OOP medical expenditure. The fact that indirect expenditure (e.g., transportation, food, lost productivity due to illness) were not included in OOP medical expenditure leads to an underestimated CHE incidence and intensity to some extent [32, 34, 43]. Thirdly, it is worth emphasizing that the analysis of the association between CHE incidence and annual per capita household income, or between CHE incidence and self-rated health status of household heads, is not a strictly causal inference, and thus the relevant descriptions in the discussion section should not be interpreted as causal associations.

## Conclusion

In conclusion, the present study identified that a certain proportion of incurring CHE existed in both urban and rural households in China, and rural households had higher risk of incurring CHE than urban households. The strong pro-poor inequality for CHE incidence and overshoot could be found in both two groups. In contrast, the problem of poverty due to illness was more severe for rural low-income groups than for urban low-income groups. Therefore, relevant policy interventions should further focus on narrowing the income gap among different groups, generally improving citizens’ education level, encouraging the development of supplementary medical insurance, and increasing the reimbursement rate for hospitalization expenses in the medical assistance system.

## Data Availability

The data source of this study was a publicly available database, the China Family Panel Studies (CFPS), which was hosted by the Institute of Social Science Survey (ISSS) of Peking University every 2 years from 2010 to 2018.
